# Unraveling the Immunomodulatory Mechanisms of Quan-du-zhong Capsule in Diabetic Kidney Disease *via* Integrated Network Pharmacology and Experimental Validation

**DOI:** 10.2174/0118715303398054250823130459

**Published:** 2025-09-25

**Authors:** Guohua Liu, Chaozhong Zhou, Xunli Xiao, Siyuan Hu, Bo Xie, Zonghai Wu, Jun Xiao

**Affiliations:** 1 Department of Pharmacy, Affiliated Hospital of Jinggangshan University, Jiangxi, 343000, Ji'an, China;; 2 Jiangxi Puzheng Pharmaceutical Co, Ltd., Jiangxi, 343100, Ji'an, China;; 3 Key Laboratory of Modern Preparation of Traditional Chinese Medicine, Ministry of Education, School of Pharmaceutical Sciences, Zhejiang Chinese Medical University, Hangzhou, 311400, China

**Keywords:** Quan-du-zhong capsule, bioinformatics, molecular docking, immunomodulatory effects, diabetic kidney disease, network pharmacology

## Abstract

**Introduction:**

Quan-du-zhong capsule (QDZ), derived from *Eucommia ulmoides* Oliv, is clinically utilized for diabetic kidney disease (DKD) management due to its renoprotective effects. Recent studies have demonstrated that QDZ ameliorates proteinuria and attenuates the decline in glomerular filtration rate (GFR) in DKD patients; however, the underlying mechanisms remain unclear.

**Methods:**

To elucidate the active components of QDZ and their potential association with immune cell modulation, we conducted bioinformatics analyses using GEO datasets and CIBERSORT to assess immune cell infiltration. Furthermore, molecular docking experiments and experimental validation were performed to verify the interactions between QDZ and potential immunotherapeutic targets.

**Results:**

Network pharmacology analysis identified the main active components of QDZ, including Quercetin, Kaempferol, β-carotene, β-sitosterol, and Syringetin. Furthermore, bioinformatics and molecular docking studies demonstrated that the FOS gene and the MAPK signaling pathway exhibit differential expression in DKD patients and were significantly correlated with immune cell activity. Notably, the active components—particularly Quercetin, Kaempferol, and Syringetin—displayed strong binding affinities to key targets. In addition, QDZ significantly upregulated FOS and MAPK expression and enhanced glucose uptake in HG-induced HEK-293 cells, suggesting its role in improving insulin sensitivity.

**Discussion:**

In our study, we indicated that FOS is acritical target in the treatment of DKD with QDZ and is closely associated with immune infiltration and the MAPK signaling pathway. Furthermore, this is the first study to validate QDZ’s immune-modulatory mechanism in DKD via the FOS-MAPK axis.

**Conclusion:**

This study illustrates the mechanism by which QDZ upregulates FOS expression and modulates the MAPK signaling pathway, thereby regulating immune cell function in DKD. These findings provide novel insights to inform future research and development of QDZ-based DKD therapies.

## 1 INTRODUCTION

DKD is one of the most common chronic microvascular complications induced by diabetes [1, 2]. In clinical practice, approximately half of patients with type 2 diabetes and one-third of patients with type 1 diabetes will develop DKD, which progressively advances to end-stage renal disease (ESRD), ultimately resulting in renal failure and uremia [3]. The traditional diagnostic criteria for DKD include persistent albuminuria and a reduced GFR, which are widely considered the classic phenotypes of the disease [4]. However, the pathogenesis of DKD is multifactorial, and the available therapeutic options remain limited [5]. DKD arises from a complex interplay between genetic predisposition and environmental factors. Recent studies demonstrate that strict blood glucose control significantly reduces the incidence of DKD [6]. This highlights the critical role of sustained hyperglycemia-induced alterations in energy metabolism and mitochondrial dysfunction in the onset and progression of DKD [6]. Additionally, controlling blood pressure fluctuations, particularly through the use of antihypertensive medications, has proven to be an effective strategy in slowing the progression of DKD [6]. Therefore, the development of new potential drugs to prevent and treat the progression of DKD remains important.

The development of traditional Chinese medicine (TCM) and its natural extracts has become a prominent research focus [7]. Currently, many TCMs have demonstrated potential therapeutic effects in regulating type 2 diabetes. Eucommia ulmoides Oliv, a traditional TCM with a long clinical history, is renowned for its benefits in kidney supplementation, blood pressure reduction, and osteoporosis improvement [8]. The QDZ, made from an extract of the whole Eucommia ulmoides Oliv plant, is clinically used to treat various types of nephropathy due to its multifaceted pharmacological actions, including blood pressure regulation, hepatorenal protection, and musculoskeletal strengthening [9]. Recent pharmacological studies suggest that QDZ can improve glomerular lesions in patients with hypertensive nephropathy and alleviate kidney damage caused by chronic hypertension [10]. Additionally, clinical data indicate that QDZ can reduce both systolic and diastolic blood pressure in nephrotic syndrome patients, contributing to the restoration of physiological blood pressure rhythms [11]. Studies have also shown that QDZ promotes angiogenesis by activating VEGFA and its downstream PI3K/AKT signaling pathway [9]. However, the complex composition and the molecular mechanisms underlying QDZ limited its clinical application and further development.

Therefore, this study aims to systematically elucidate the bioactive compounds, potential targets, and molecular mechanisms of QDZ in the treatment of DKD using network pharmacology methods. Unlike previous studies, our work integrates high-throughput gene expression analysis with molecular docking and cellular validation to establish FOS as a potential immune-regulatory target in DKD. Furthermore, this represents the first demonstration of the immunomodulatory mechanism of QDZ through the FOS-MAPK signaling axis in DKD. The latest research indicates that the immunomodulatory pathway is a therapeutic strategy for DKD [12, 13]. FOS is the protein product of the c-fos proto-oncogene and is related to immunoreactivity [14, 15]. Furthermore, MAPK also has *in vivo* immunomodulatory activity [16, 17]. These findings suggested that FOS and MAPK may have the potential to be immunomodulatory targets and pathways for improving immune function in DKD. Bioinformatics analysis of clinical DKD sample data will be employed to validate the network pharmacology results, and molecular docking experiments will be conducted to further verify the targets of QDZ in treating DKD. Through comprehensive analysis and validation, this study will uncover the targets and molecular mechanisms underlying the therapeutic effects of QDZ on DKD, providing evidence for future clinical applications.

## 2 METHODS

### 2.1 Chemicals and Reagents

QDZ was sourced from Jiangxi Puzheng Pharmaceutical Co., Ltd. (Approval No: State Pharmaceutical License Z20055116, Production Batch No: 240202). The CCK-8 cell viability assay kit was obtained from Abcam (Cambridge, UK). RNA extraction was performed using a kit from Aidlab Biotech (Beijing, China). The iScript cDNA synthesis kit and SYBR Green master mix were purchased from Vazyme Biotech (Nanjing, China). Antibodies targeting FOS (#2250), β-actin (#4967), and secondary antibodies were acquired from Cell Signaling Technology (Danvers, MA, USA). The bicinchoninic acid (BCA) assay kit and the Western Lightning™ Plus-ECL enhanced chemiluminescence substrate assay kit were supplied by Thermo Scientific (Grand Island, NY, USA).

### 2.2 Screening of Active Compounds and Targets of QDZ

QDZ is a monotherapy formulation composed of the traditional Chinese medicine Eucommia ulmoides Oliv. The chemical components of Eucommia ulmoides Oliv in QDZ were identified through the Traditional Chinese Medicine Systems Pharmacology (TCMSP) database and analysis platform. Active compounds with oral bioavailability (OB) ≥ 30% and drug-likeness (DL) ≥ 0.18 were selected as potential bioactive ingredients. Relevant targets of the active compounds were retrieved from the TCMSP database, and the protein targets obtained were standardized using the Uniprot database (https://www.uniprot.org/), where target names were converted into their corresponding symbol names.

### 2.3 Screening of Targets Related to DKD

Target screening for DKD: DKD-related targets were identified from the GeneCards database (https://www.genecards.org/), OMIM database (https://www.omim.org/), and PharmGKB database (https://www.pharmgkb.org/). A Venn diagram was constructed using R 4.4.0 software to visualize the overlap between QDZ and DKD targets. Common targets were then used as selection criteria to identify the major bioactive components associated with QDZ. Additionally, Cytoscape 3.10 software was utilized to construct a drug-component-target-disease network diagram, based on the relationships between the intersecting targets, diseases, and the active components of traditional Chinese medicine.

### 2.4 Construction of the PPI Network

A total of 132 intersecting targets were uploaded to the STRING database (version: 12.0) (https://cn.string-db.org/) to generate a protein-protein interaction (PPI) network for “Homo sapiens” [18]. The results from the STRING database were visualized using Cytoscape 3.10 software, and network topology parameters (degree, betweenness, eigenvector, and closeness) of the intersecting targets were obtained using the NetworkAnalyst tool within Cytoscape. The genes with scores higher than the median values of network topology parameters were regarded as core targets [19]. The active ingredients of QDZ and core targets were imported into the Cytoscape 3.10 software to construct the network of the bioactive compounds and core targets of QDZ [20].

### 2.5 GO and KEGG Enrichment Analysis of the Intersecting Targets Between QDZ and DKD

GO and KEGG enrichment analyses were conducted using the “clusterProfiler” and “enrichplot” R packages, installed from the Bioconductor database (version: 3.19) (https://bioconductor.org/) into R 4.4.0 software. Representative clusters and pathways were selected with a threshold of p ≤ 0.01 from both GO and KEGG analyses. Enrichment results were visualized through bubble plots, bar plots, and corresponding pathway diagrams using the “ggplot2” R package.

### 2.6 Collection and Analysis of Public GEO Data

The data for this study were obtained from the GEO database (http://www.ncbi.nlm.nih.gov/gds/) [21]. The GSE96804 dataset includes mRNA data from 41 DKD patients and 20 healthy controls [22, 23]. Differential analysis was performed using the “limma” R package [24]. Using the “NormalizeBetweenArrays” function, data were normalized [25]. Then, a linear model of the data was constructed using the “lmFit” function, and the correlation coefficient and standard deviation were calculated using the “eBayes” function [26]. Differentially expressed genes (DEGs) were identified using *p* < 0.05 and |logFC| > 0.585 as selection criteria. The DEGs were visualized using volcano plots and heatmaps generated by the “ggplot2” and “pheatmap” R packages.

### 2.7 Bioinformatics Analysis of Clinical DKD Data

To explore the potential pathogenesis of DKD, gene set enrichment analysis (GSEA) (version: 4.1.0) was conducted on the DEGs using the “clusterProfiler” R package, and visualization was performed using the “enrichplot” R package. To determine the enrichment scores for each sample in the gene set, the method described by Hanzelmann *et al*. was adopted, and molecular features were downloaded from the Molecular Signatures Database (MSigDB) (version: 7.4) using the c2.cp.kegg.v7.4.symbols.gmt gene set [27]. The enrichment scores for each sample in the gene set were calculated [27, 28]. This process resulted in the creation of an enrichment score matrix. The FDR for DEGs was calculated using the p.adjust function, and pathway activity scores were analyzed using the “gplots” R package. Pearson correlation coefficients between FOS genes and the MAPK signaling pathway were computed using the “cor” function in R, based on pathway activity scores and gene profiles across samples [29].

### 2.8 Identification and Correlation Analysis of Immune Infiltrating Cells in DKD

Immune cell infiltration plays a crucial role in predicting disease progression and therapeutic response [7]. To estimate immune cell infiltration based on gene expression profiles, we used the “preprocessCore” package and the leukocyte gene signature set (LM22) [30]. In this study, the CIBERSORT algorithm implemented in R software (version 4.4.0) was used to calculate the composition of 22 different immune cell types in glomerular samples from DKD patients, aiming to identify potential immune therapy targets [31]. The sum of the estimated proportions of all immune cell types in each sample is equivalent to 1 [32]. The relative proportions of the 22 immune cells in each sample were quantified using the expression data from the GSE96804 cohort. A box plot was used to visualize the distribution of immune cell populations in the DKD group. To assess the differential changes based on FOS, the differences in immune cell proportions between the DKD and control groups were evaluated using a t-test, with a significance level set at 0.05.

### 2.9 Molecular Docking of Core Targets and Major active Components

The three-dimensional crystal structures of core targets were downloaded from the Protein Data Bank (PDB) (https://www.rcsb.org/), which needs to meet the requirement that the RMSD value is less than 2 [33], and the 3D structures of major active components were downloaded from the PubChem database (https://pubchem.ncbi.nlm.nih.gov/). Their pdb format files were downloaded, de-watered, and hydrogenated in AutoDockTools (version: 1.5.7), and then selected as receptors and saved as pdbqt files [34]. The mol2 files of the active components were obtained through the TCMSP database, hydrogenated in AutoDockTools, selected as ligand and exported as pdbqt files [34]. The standard ligand was redocked to the protein using AutoDockTools 1.5.7 with the AutoDock4 method for validation [35]. In the docking process, the grid box size was 70 × 70 × 70 Å^3^ with x, y, and z coordinates of 26.45, 8.595, and -15.21, respectively. Molecular docking was conducted using AutoDock software (version: 1.2.3) [36, 37], and the affinity of major active components for core targets was visualized through heatmaps generated using the “pheatmap” R package. Active components and targets with high binding affinity were selected and visualized using PyMOL software (version 2.6.0).

### 2.10 Cell Culture

The HEK-293 cells were obtained from Fenghui Biotechnologies, Inc. (Changsha, China). Both cell lines were confirmed to be free of mycoplasma and were authenticated using the short tandem repeat (STR) method, demonstrating high genetic consistency. The cells were cultured in low-glucose DMEM (LG) medium (Gibco, Grand Island, NY, USA), supplemented with 10% fetal bovine serum (FBS, Gemini, Grand Island, NY, USA), and maintained in a humidified incubator at 37°C with 5% CO_2_.

### 2.11 DKD Cellular Model Establishment and Cell Viability Assay

Common podocytes, or mesangial cells may better mimic DKD glomerular pathology. However, Many published studies have also used HG to induce HEK-293 cells to establish *in vitro* DKD cell models [38-40]. To create an *in vitro* cellular model of DKD, HEK-293 cells were cultured in high-glucose DMEM (HG) containing 30 mmol/L glucose, 10% fetal bovine serum (FBS), and 1% penicillin-streptomycin. Cell viability was assessed using the CCK-8 assay. In brief, HEK-293 cells were plated in 96-well plates at a density of 1 × 10^4^ cells /well. Once the cells reached approximately 50% confluence, they were treated with QDZ at concentrations of 25, 50, or 100 μg/mL for 24 hours. Following treatment, 20 μL of CCK-8 solution was added to each well, and the plates were incubated for 4 hours. Absorbance at 450 nm was measured using a Cytation 5 microplate reader (BioTek, USA). In addition, HEK-293 cells were cultured in DMEM with 24.5 mmol/L mannitol and 5.5 mmol/L glucose, which was used to control cell osmolality.

### 2.12 Quantitative Real-time PCR (qRT-PCR)

To evaluate mRNA expression, HEK-293 cells were seeded in 6-well plates at a density of 2 × 10^5^ cells/well. Once the cells reached 50% confluence, they were treated with or without QDZ for 24 hours. Total RNA was extracted using an RNA extraction kit according to the manufacturer’s instructions. The quality and quantity of the RNA were assessed using a NanoDrop 2000c spectrophotometer (Thermo Scientific, Grand Island, NY, USA). One microgram of total RNA was reverse transcribed to cDNA using the iScript cDNA synthesis kit, following the manufacturer’s guidelines. qRT-PCR was performed with SYBR Green master mix on a Bio-Rad real-time PCR system. The PCR cycling conditions included denaturation at 95°C for 5 seconds, annealing at 60°C for 30 seconds, and extension at 65°C for 5 seconds, for a total of 40 cycles. Relative gene expression was calculated using the 2^-ΔΔ-Ct^ method [41], with β-actin serving as the internal control. The primer sequences used for amplification were as follows: hFOS forward, 5’-CCGGGGATAGCCTCTCTTACT-3’ and reverse, 5’-CCAGGTCCGTGCAGAAGTC-3’; hMAPK forward, 5’-TACACCAACCTCTCGTACATCG-3’ and reverse, 5’-CATGTCTGAAGCGCAGTAAGATT-3’; hβ-actin forward, 5’-CTGGAACGGTGAAGGTGACA-3’ and reverse, 5’-AAGGAACTTCCTTGAACAATGCA-3’.

### 2.13 Western Blot Analysis

To evaluate protein expression, HEK-293 cells were cultured in 6-well plates (2 × 10^5^ cells/well). Upon reaching 50% confluence, the cells were treated with or without QDZ for 24 hours. Following treatment, the cells were lysed with 100 μL of ice-cold RIPA buffer, supplemented with protease and phosphatase inhibitors, using ultrasonication. After centrifugation at 4 °C, the protein concentration in the lysate was measured using a BCA assay kit according to the manufacturer's instructions [42]. A total of 30 μg of protein was separated by SDS-PAGE and transferred onto a polyvinylidene fluoride (PVDF) membrane. The membrane was blocked with 5% skim milk in Tris-buffered saline with Tween-20 (TBST) for 1 hour at room temperature (RT), followed by overnight incubation with primary antibodies (1:1000 dilution) in 5% skim milk at 4 °C in TBST [42]. The membranes were washed at least three times for 10 minutes each in TBST, then incubated with secondary antibodies (1:2000 dilution) for 1 hour at RT. After three additional washes in TBST, protein bands were detected using an enhanced chemiluminescence (ECL) substrate. The images were captured using the Minichemi™ 610 Chemical Imaging System (Beijing, China), and the relative optical density of the bands from three independent experiments was analyzed using ImageJ software [42].

### 2.14 Statistical Analysis

All data have been presented as the M±SD of multiple samples. ANOVA and Bonferroni post hoc correction were used for univariate multiple comparisons. Two-way ANOVA was used for multiple comparisons of two variables, followed by Bonferroni post hoc tests. a result of *P*<0.05 was deemed statistically significant.

## 3 RESULTS

### 3.1 Screening of Active Ingredients and Target Proteins in QDZ

Active ingredients in QDZ were screened using the TCMSP database platform, with OB ≥ 30% and DL ≥ 0.18. The corresponding target proteins for these active ingredients were converted and deduplicated using the UniProt database, resulting in 132 unique gene targets. Disease-related genes were obtained from several databases: 3,814 genes from the GenCards database (with a score > 1), 97 genes from the OMIM database, and 58 genes from the PharmGKB database. After deduplication and integration, a total of 3,873 DKD target genes were identified, as shown in Fig. (**1A**). A Venn diagram of QDZ and DKD targets was generated using R 4.4.0 software, as depicted in Fig. (**1B**), revealing 132 common targets shared between the herbal medicine and the disease.

The common targets of the disease and QDZ were further analyzed. The corresponding relationships between active ingredients and their targets, as well as between targets and diseases, were imported into Cytoscape 3.10 software for network construction. The drug, its active ingredients, targets, and related diseases were optimized to form a drug-component-target-disease network. In this network, two nodes are connected by an edge if they are related. The more edges a node has, the higher its degree value, indicating its greater importance in the network, as illustrated in Fig. (**1C**). Using Cytoscape’s Network Analyzer, the degree values of active ingredients and genes in QDZ were calculated. Based on the target selection, 23 active ingredients containing corresponding targets were identified. The top five active ingredients most strongly associated with the targets were Quercetin, Kaempferol, β-carotene, β-sitosterol, and Syringetin, as shown in Table (**1**).

### 3.2 Construction of PPI Network

The 132 common intersecting targets of drugs and diseases were imported into the STRING database to construct the PPI network. The confidence score was set to 0.9, and the free-standing targets were excluded. The resulting network consisted of 132 nodes and 2,977 edges. The PPI network diagram and corresponding TSV file for the target genes were exported, as shown in Fig. (**2A**). To better illustrate the importance of each target in the PPI network, the TSV file was imported into Cytoscape to calculate the degree values of each target. The PPI network was then visualized based on the degree values, as illustrated in Fig. (**2B**). Furthermore, the orange-colored targets in the network represent those with greater influence, as illustrated in Fig. (**2B**). (Fig. **2C**) presents the top 20 genes ranked by their degree values. Through analysis using the CytoNCA plugin, we identified nine core genes (TP53, ESR1, AKT1, CCND1, MAPK1, HSP90AA1, FOS, CTNNB1, and BCL2), as depicted in Fig. (**2D**) [43]. Furthermore, relevant literature reports that the top-ranked core genes are involved in the regulation of kidney diseases and are also closely related to immunotherapy [44-47]. In summary, this analysis provides valuable insights into the potential mechanisms of QDZ in the treatment of DKD.

### 3.3 GO Function and KEGG Pathway Enrichment Analysis

GO and KEGG pathway analyses were conducted using the respective R packages from the Bioconductor database, yielding the major GO functional bar chart and KEGG pathway enrichment bubble chart, as shown in Figs. (**3A**-**B**). The GO functional analysis was categorized into three main aspects: Biological Processes (BP), Cellular Components (CC), and Molecular Functions (MF). The clustering analysis results for GO-BP are presented in Fig. (**3A**), highlighting five representative clusters: response to xenobiotic stimulus, response to molecules of bacterial origin, response to lipopolysaccharide, response to reactive oxygen species, and response to oxidative stress. For GO-CC, the clustering analysis identified five key clusters: membrane raft, membrane microdomain, protein kinase complex, cyclin-dependent protein kinase holoenzyme complex, and RNA polymerase II transcription regulator complex. The GO-MF clustering analysis revealed five major clusters, including DNA-binding transcription factor binding, RNA polymerase II-specific DNA-binding transcription factor binding, nuclear receptor activity, ligand-activated transcription factor activity, and transcription coregulator binding.

KEGG pathway enrichment analysis ranked the pathways from high to low, with the PI3K-Akt signaling pathway, AGE-RAGE signaling pathway, MAPK signaling pathway, TNF signaling pathway, IL-17 signaling pathway, apoptosis, toll-like receptor signaling pathway, HIF-1 signaling pathway, NOD-like receptor signaling pathway, FoxO signaling pathway, Ras signaling pathway, NF-κB signaling pathway, PD-L1 signaling pathway, JAK-STAT signaling pathway, and insulin resistance pathway being identified, as illustrated in Fig. (**3B**). Based on the traditional Chinese medicine-ingredient-target-disease network, PPI protein interactions, and KEGG pathway enrichment results, the MAPK signaling pathway was determined to be the central pathway, and the corresponding pathway diagram was generated, illustrating its key role in the pathogenesis of DKD, as displayed in Fig. (**3C**).

### 3.4 Differentially Expressed Genes in glomeruli of DKD Patients and GSEA analysis

To investigate the gene expression alterations in the glomeruli of DKD patients, we retrieved the gene expression dataset GSE96804 from the GEO database. The GSE96804 dataset, collected from the GPL17586 microarray platform, contains gene expression profiles from patients with DKD. This dataset includes glomeruli from 20 healthy individuals and 41 patients with DKD. Differential expression analysis revealed that, compared to the healthy group (Normal group), the DKD group exhibited 993 upregulated and 870 downregulated genes, for a total of 1863 differentially expressed genes, as shown in Fig. (**4A**). We annotated the 9 core targets identified in network pharmacology on a volcano plot, and found that only the FOS gene was significantly downregulated in the DKD patient group, while other core genes did not show significant changes, as illustrated in Fig. (**4A**). Additionally, a heatmap representation of the 9 core targets is shown in Fig. (**4B**). As depicted in Fig. (**4C**), FOS gene expression was significantly downregulated in the DKD patient group compared to the Normal group.

To further identify the activated signaling pathways associated with differential gene expression in DKD patients, we performed GSEA analysis. The GSEA results revealed that the MAPK signaling pathway was significantly enriched in the DKD patient group, as shown in Fig. (**4D**), which is consistent with the aforementioned KEGG enrichment results. Furthermore, as shown in Fig. (**4E**), the MAPK signaling pathway was significantly downregulated in the DKD patient group compared to the Normal group. Additionally, correlation analysis indicated a positive correlation between the expression of the core target FOS and the MAPK signaling pathway, as shown in Fig. (**4F**).

### 3.5 The relationship between DKD and Immune Cells

Immune cell infiltration analysis plays a crucial role in predicting disease progression and treatment response [7]. To investigate the changes in immune cells in the glomeruli of DKD patients, we analyzed the expression profiles of 22 immune cell types in DKD samples from the GSE96804 dataset and calculated their scores using the tumor immunology tool CIBERSORT [7]. Inflammation plays a key role in the pathogenesis of DKD, and immune cells act as mediators of inflammation in diabetic nephropathy [48]. Immune cells such as Macrophages and T lymphocytes are the main immune cells associated with renal injury [49, 50]. Furthermore, the FOS gene is related to the inflammatory response [51]. As shown in Fig. (**5A**), we assessed the infiltration of immune cells and evaluated the expression levels of immune cells in the DKD group. Compared to other immune cell types, the DKD group exhibited a higher proportion of T cells CD4 memory resting, activated NK cells, Monocytes, M1 macrophages, and M2 macrophages. Furthermore, we also examined the correlation between the core gene FOS and immune cells. As illustrated in Fig. (**5B**), red dots indicate a negative correlation between the core gene and immune cells, while green dots indicate a positive correlation, with the size of the dots reflecting the strength of the correlation. These findings open up potential avenues for the development of therapeutic interventions targeting immune cells related to FOS.

### 3.6 Molecular Docking Validation

Based on the above analysis, we identified five core components of QDZ (Quercetin, Kaempferol, β-carotene, β-sitosterol, and Syringetin) and the key target FOS. Using AutoDock software, we performed molecular docking between the five core components and FOS, with the docking results presented in a heatmap, as shown in Fig. (**6A**). An affinity < −4.25 kcal/mol indicates binding activity between the ligand and the target, while an affinity < −5.0 kcal/mol suggests good binding activity, and an affinity < −7.0 kcal/mol indicates strong docking activity [52]. We found that the docking affinities of Quercetin, Kaempferol, and Syringetin with FOS were −10.4, −9.7, and −8.5 kcal/mol, respectively, demonstrating strong binding activity, as illustrated in Fig. (**6A**). Using PyMOL software, we visualized the docking results of these three compounds, as displayed in Figs. (**6B**-**D**), where hydrogen bonds and their lengths are displayed, along with the amino acid residues connected to the ligands. For example, the docking of FOS and Quercetin, which exhibited the lowest binding energy of −10.4 kcal/mol, involved eight hydrogen bonds. The amino acid residues interacting with Quercetin were DA-9, DA-31, DG-8, DG-10, DG-30, SER-278, and ARG-155. These results suggest that Quercetin, Kaempferol, β-carotene, β-sitosterol, and Syringetin in QDZ play a significant role in the therapeutic effects on DKD through the FOS target, providing a theoretical basis for studying the mechanisms of action of QDZ in DKD treatment.

### 3.7 QDZ Upregulates the Expression of FOS and MAPK Signaling Pathways to Promote Glucose Uptake in HG-induced HEK-293 Cells

To determine whether HG and high osmotic stress (CON-M) have toxic effects on the cells, we evaluated the cell viability of HEK-293 cells after 24 hours of induction treatment with HG medium and mannitol isotonic medium. As shown in Fig. (**7A**), we found that the HG medium and CON-M had no cytotoxic effect on HEK-293 cells. To assess the effects of different concentrations of QDZ (25–200 µg/mL) on the viability of HG-induced HEK-293 cells, cell viability was evaluated after 24 hours of QDZ treatment at various concentrations. As shown in Fig. (**7A**), no significant decrease in cell viability was observed in any of the treatment groups compared to the control group. Notably, treatment with 200 µg/mL QDZ even promoted cell growth. These results indicate that QDZ at concentrations ranging from 25 to 200 µg/mL did not exhibit notable cytotoxicity in HEK-293 cells. Based on these findings, QDZ concentrations of 25, 50, and 100 µg/mL were selected for subsequent experiments.

To simulate the renal insulin resistance phenotype in DKD patients, HEK-293 cells were treated with 30 mM HG for 24 hours, which is a commonly used *in vitro* model for kidney insulin resistance [38-40]. As expected, glucose uptake experiments revealed a significant reduction in glucose uptake in HEK-293 cells following HG treatment, as illustrated in Fig. (**7B**). Furthermore, compared with the CON group, there was little change in glucose uptake in the CON-M group, indicating that high osmotic stress had no effect on cellular glucose uptake in Fig. (**7B**). In contrast, treatment with various concentrations of QDZ (in a dose-dependent manner) significantly enhanced glucose uptake, as displayed in Fig. (**7B**). To further validate the results from network pharmacology and bioinformatics analyses, the expression of FOS and MAPK genes was assessed. In HG-treated HEK-293 cells, both FOS and MAPK gene expression were significantly reduced at the mRNA level, as displayed in Figs. (**7C**-**D**). However, compared to the HG group, treatment with different concentrations of QDZ (in a dose-dependent manner) significantly increased the expression of both FOS and MAPK genes. Moreover, in HG-treated HEK-293 cells, QDZ treatment also significantly upregulated FOS protein expression in a dose-dependent manner, as shown in Fig. (**7E**). In summary, these findings suggest that in the HG-treated HEK-293 cell model, QDZ upregulates the expression of FOS and MAPK genes, thereby enhancing glucose uptake and potentially improving DKD.

## 4 DISCUSSION

DKD is a common microvascular complication and a leading cause of death among diabetic patients, imposing a substantial global economic burden [53, 54]. However, the exact pathogenesis of DKD remains unresolved. Current research indicates that immune cells and immune cell-derived factors can accelerate the progression of DKD through various mechanisms [55]. The kidneys are composed of diverse, highly differentiated cell types, with different immune cells playing crucial regulatory roles in the progression of renal diseases [55]. Currently, drugs used to treat DKD have limitations due to their narrow therapeutic scope, significant adverse effects, so the clinical risks and benefits need to be further evaluated. Traditional Chinese medicine, characterized by low toxicity, multi-target action, and broad application potential, is gradually gaining attention as a promising treatment option.

QDZ exemplifies the characteristic multi-component, multi-target, and multi-pathway therapeutic approach of traditional Chinese medicine (TCM), where a single active ingredient can modulate multiple targets, while each target may influence various biological processes and signaling pathways. Therefore, this is highly beneficial for the treatment of complex diseases such as DKD. Through network pharmacology analysis, we identified five key bioactive compounds in QDZ, including Quercetin, Kaempferol, β-carotene, β-sitosterol, and Syringetin. Among them, Quercetin, Kaempferol, and β-sitosterol exhibit notable antioxidant, anti-inflammatory, nephroprotective, and anti-diabetic properties, which can effectively improve the pathological symptoms of DKD [56-59]. Clinical studies have shown that dasatinib combined with quercetin has a good therapeutic effect on DKD [60]. Furthermore, Liu *et al*. demonstrated that quercetin could prevent DKD by inhibiting renal tubular epithelial cell apoptosis through the PI3K/AKT pathway [61]. Research by Alshehri indicated that Kaempferol alleviates proteinuria in diabetic rats through its hypoglycemic effect, along with the activation of the Nrf-2/HO-1/antioxidant axis [58]. β-sitosterol exerts anti-inflammatory, antioxidant, and anti-apoptotic activities in high-glucose-induced podocytes and HK-2 cells by inhibiting the activation of the TLR4/NF-κB signaling pathway [59]. β-carotene modulates autophagy in renal tissues of diabetic rats through the AMPK/SIRT1 pathway, reducing inflammatory responses [62]. Additionally, Syringetin demonstrates multiple pharmacological activities, including antitumor, hepatoprotective, anti-diabetic, and antioxidant effects [63]. A study by Wu *et al*. revealed that Syringetin, as an α-glucosidase inhibitor, can reduce postprandial blood glucose by inhibiting carbohydrate absorption, thus showing promise for treating diabetes [64].

FOS, a proto-oncogene widely expressed in eukaryotic genomes, exhibited increased expression in response to various external stimuli, including neurotransmitters, serving as a marker of neuronal activation [65]. Additionally, recent studies have suggested that FOS may both serve as a potential diagnostic biomarker and, when combined with emetine, a promising therapeutic agent for DKD [51]. In this study, network pharmacology and clinical sample biological analyses predicted that FOS is a core target of QDZ in DKD treatment. Moreover, network pharmacology identified the key active components of QDZ in the treatment of DKD, which include Quercetin, Kaempferol, β-carotene, β-sitosterol, and Syringetin. Molecular docking confirmed that the major active components strongly bind to FOS, reflecting the reliability of the network analysis. Among these interactions, the binding activities of Quercetin-FOS, Kaempferol-FOS, and Syringetin-FOS were relatively stronger, but further experimental validation is needed. A key regulatory element is present within the FOS protein, and its phosphorylation, mediated by the MAPK signaling pathway, contributes to protein stability [66, 67]. GSEA and KEGG enrichment analyses showed a significant downregulation of the MAPK signaling pathway in the DKD patient group. Furthermore, correlation analysis revealed a positive correlation between the expression of the core target FOS and the MAPK signaling pathway. The role of the MAPK pathway in DKD was controversial. Some studies have shown that some Chinese herbs (such as apigenin, Huangkui capsule) have inhibitory effects on MAPK activity and can effectively reduce or even prevent the occurrence of DKD [68, 69], while others showed that some Chinese herbs (such as Ginsenoside Rg3) can induce the activation of the MAPK pathway [70-72]. For example, Chen *et al*. revealed that Rg3 could activate the MAPK pathway by downregulating miR-216a-5p in HG-induced SV40 MES 13, and Rg3 or miR-216a-5p inhibition could also induce the MAPK pathway in kidney tissues of diabetic model mice [70]. In addition, Rg3 can also induce angiogenesis in human endothelial cells and enhance natural killer cell activity through the MAPK/ERK pathway [71, 72]. In our study, we also revealed that QDZ significantly upregulated FOS and MAPK expression and enhanced glucose uptake in HG-induced HEK-293 cells, suggesting its role in improving insulin sensitivity and immunomodulatory Mechanisms. However, the specific mechanism still needs further verification.

According to reports, neutrophils play a significant role in the development and progression of diabetes [73]. In diabetic patients, the number of neutrophils is significantly increased, and they release various inflammatory mediators and cytokines, thereby recruiting macrophages and other immune cells, including T cells and NK cells [74, 75]. Therefore, the immune regulation of neutrophils could serve as a potential intervention strategy for diabetes-related diseases. In this study, we analyzed samples from patients with DKD and found that the proportions of T cells CD4 memory resting, activated NK cells, Monocytes, M1 macrophages, and M2 macrophages were significantly higher in the DKD group compared to other immune cells. Furthermore, these immune cells showed a linear correlation with the core gene FOS. Based on these findings, we indicated that FOS is a critical target in the treatment of DKD with QDZ and is closely associated with immune infiltration and the MAPK signaling pathway. Furthermore, this is the first study to validate QDZ’s immune-modulatory mechanism in DKD via the FOS-MAPK axis.

In our study, we found that QDZ significantly upregulated FOS and MAPK expression and enhanced glucose uptake in HG-induced HEK-293 cells, suggesting its role in improving insulin sensitivity. However, this study utilized HEK-293 cells, which are not specialized renal cells; future work should validate findings in podocytes or *in vivo*. Furthermore, although bioinformatics analysis indicated a potential association between the FOS gene and the MAPK signaling pathway, this supposed regulatory interaction needs to be further experimentally validated through subsequent mechanistic studies. We also need to conduct pharmacokinetic/pharmacodynamic (PK/PD) validation of active compounds.

## CONCLUSION

In conclusion, this study identifies FOS as a potential central biomarker and immune therapeutic target in DKD. For the first time, molecular docking and bioinformatics analyses confirm that the key active components of QDZ—Quercetin, Kaempferol, β-carotene, β-sitosterol, and Syringetin—can exert immune regulatory effects by modulating the FOS and MAPK signaling pathways, thereby inhibiting the onset and progression of DKD. This study provides valuable insights into the molecular mechanisms underlying QDZ's impact on the immune microenvironment of DKD and supports FOS as a potential target for QDZ-based DKD therapy. The continuous and in-depth research on the potential of QDZ against DKD through clinical studies using human kidney samples will undoubtedly contribute to further identifying the immunomodulatory mechanism of QDZ against DKD in future studies. These findings provide a foundational framework for developing QDZ-based interventions for immune-related renal dysfunction in diabetic patients.

## Figures and Tables

**Fig. (1):**
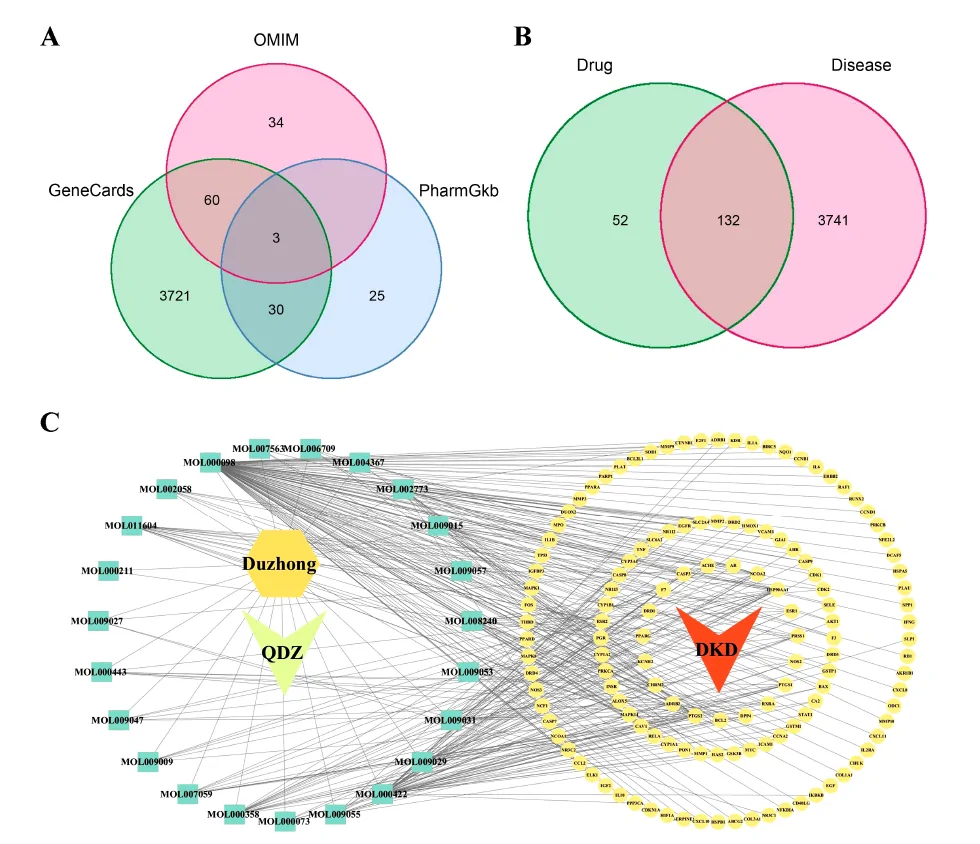
QDZ active ingredients, target screening, and network relationship construction in DKD. **(A)** Venn diagram of DKD-related targets from three disease databases; **(B)** Venn diagram of targets shared between DKD and QDZ; **(C)** Relationship mapping of 23 active ingredients and 132 common targets, generated using Cytoscape 3.10 software. The more edges there are, the more important it is. The hexagons represent traditional Chinese medicines in the formula, the green V-shaped nodes represent drugs, the red V-shaped nodes represent diseases, and the yellow ellipses represent drug-disease common genes.

**Fig. (2):**
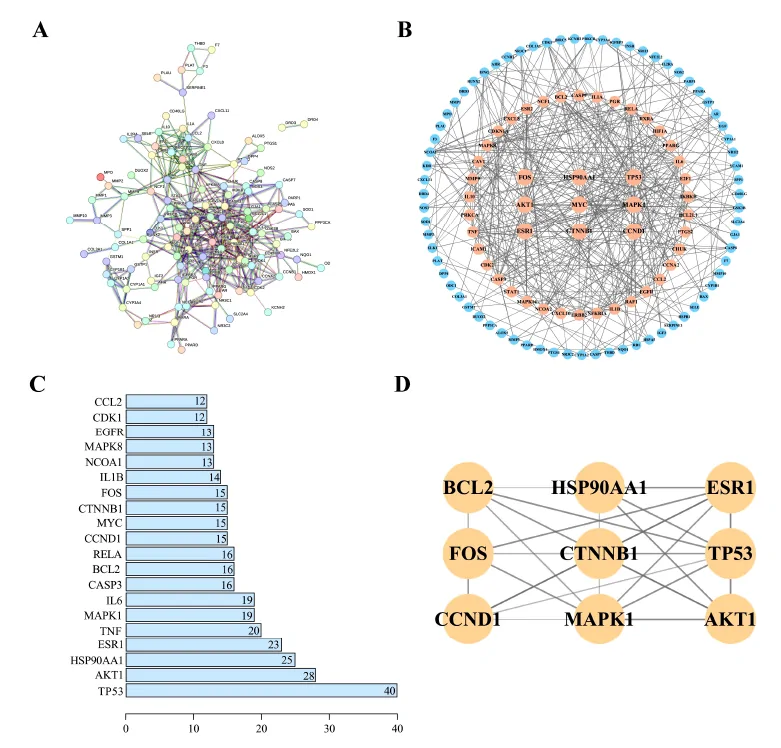
Construction and analysis of the PPI network. **(A)** Interaction network of targets shared between DKD and QDZ; **(B)** PPI network analysis of common targets between QDZ and DKD, generated using the CytoNCA plugin; **(C)** Bar chart depicting the top 20 genes with the highest interaction degrees among the intersecting genes. The horizontal axis represents the degree number, and the vertical axis represents the gene name; **(D)** Core target network of QDZ in the treatment of DKD, generated using Cytoscape software. The more edges there are, the more important it is.

**Fig. (3):**
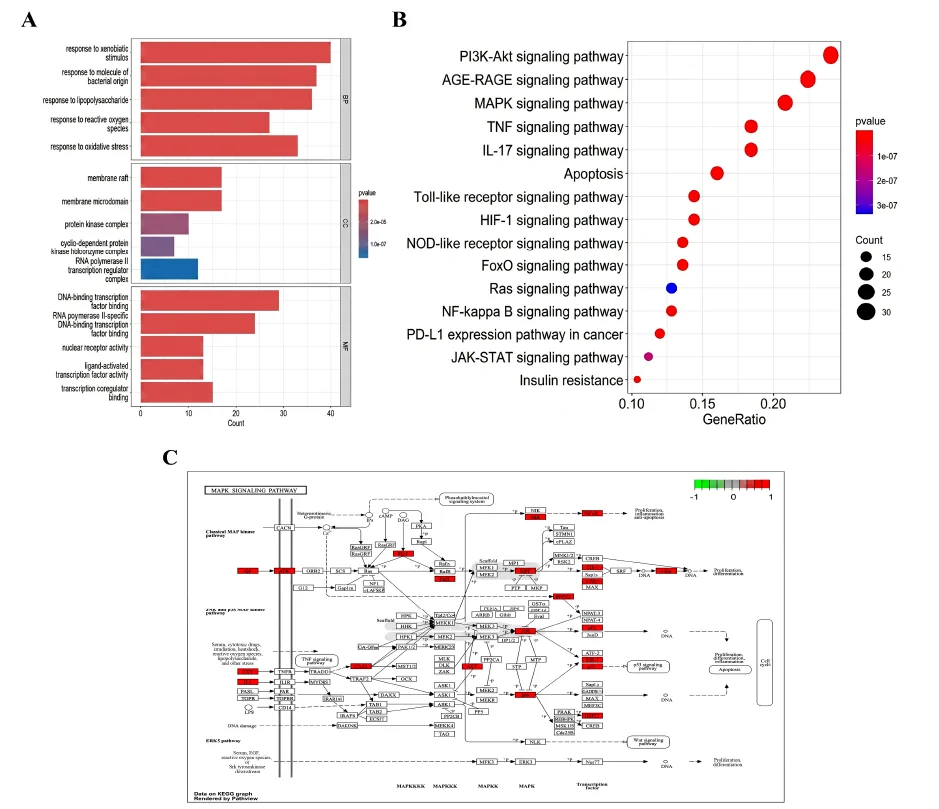
GO functional and KEGG pathway enrichment analysis of intersecting genes. **(A)** GO enrichment analysis for biological processes, molecular functions, and all components (the x-axis represents the number of genes, and the p-value is indicated by color); **(B)** KEGG pathway enrichment analysis for core targets of QDZ in the treatment of DKD (the y-axis represents pathways, the x-axis represents the number of genes, and the p-value is indicated by color); **(C)** MAPK signaling pathway (core targets of QDZ in the treatment of DKD are highlighted in red).

**Fig. (4):**
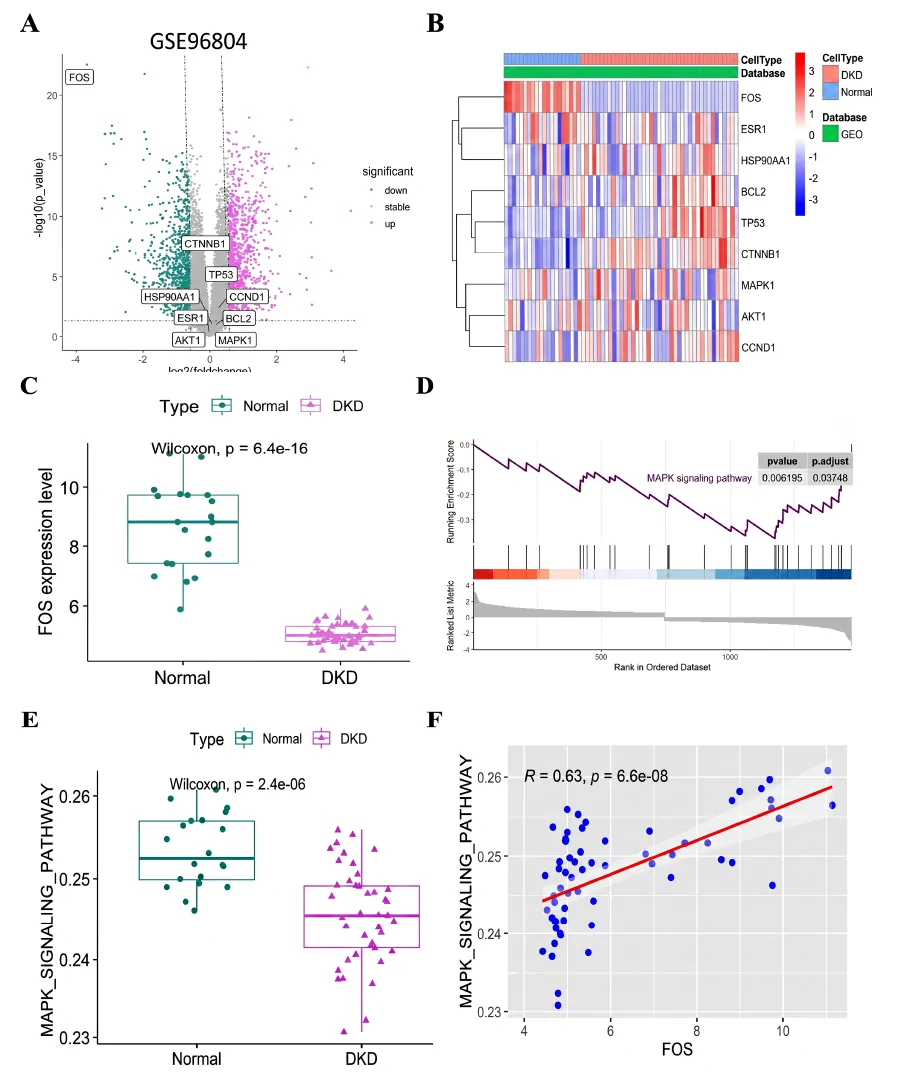
Differentially expressed genes, GSEA analysis, and correlation analysis in the glomeruli of DKD patients. **(A)** Volcano plot of differentially expressed genes between DKD patients and healthy controls (Normal) in the GSE96804 dataset; **(B)** Heatmap of the expression of 9 core genes, with the depth of the color representing the magnitude of gene changes. Red represents up-regulated genes, and blue represents down-regulated genes; **(C)** Expression of the FOS gene in the DKD patient group and the Normal group; **(D)** GSEA functional enrichment plot for the MAPK signaling pathway gene set; **(E)** Expression of the MAPK signaling pathway in the DKD patient group and the Normal group; **(F)** Correlation between FOS expression and MAPK signaling pathway expression.

**Fig. (5):**
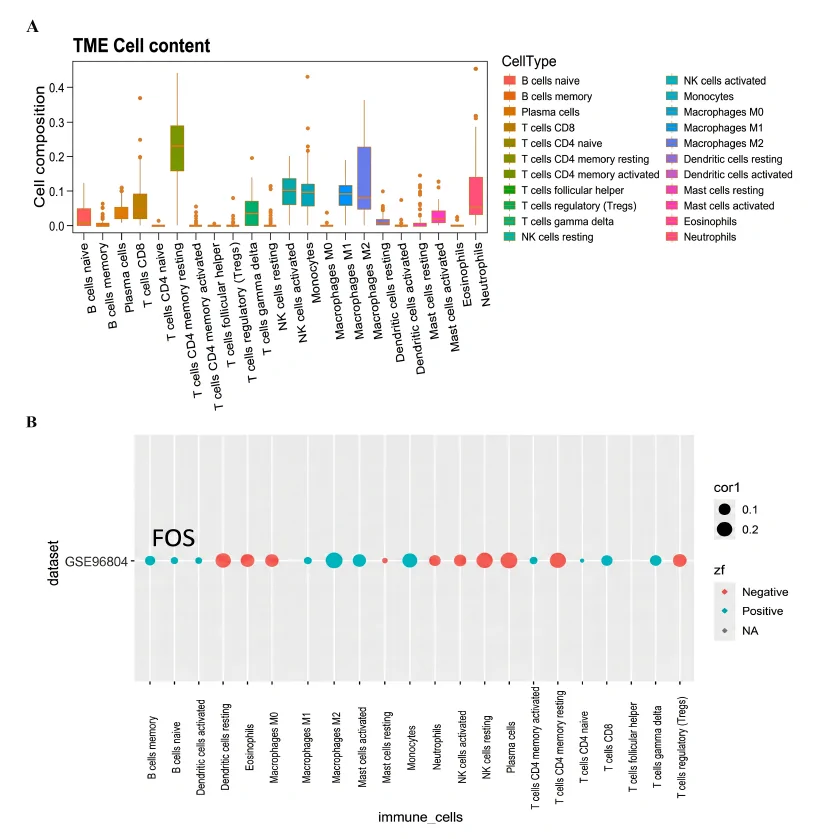
Immune characteristics of DKD. **(A)** Expression changes of different immune cell subpopulations in DKD. **(B)** Correlation between core genes FOS and various immune cell populations in DKD.

**Fig. (6):**
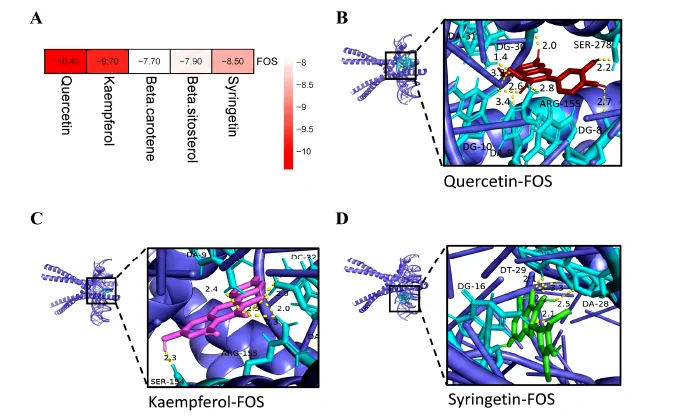
The results of molecular docking between the FOS gene and the core component. **(A)** A heat map illustrating the molecular docking results, with the depth of the color representing the intensity of the binding energy activity. The horizontal axis represents different components ** (B)** Quercetin and FOS molecular docking results (affinity: −10.4); **(C)** Kaempferol docking results with FOS (affinity: −9.7); **(D)** Results of the docking of Syringetin with FOS (affinity: −8.5).

**Fig. (7):**
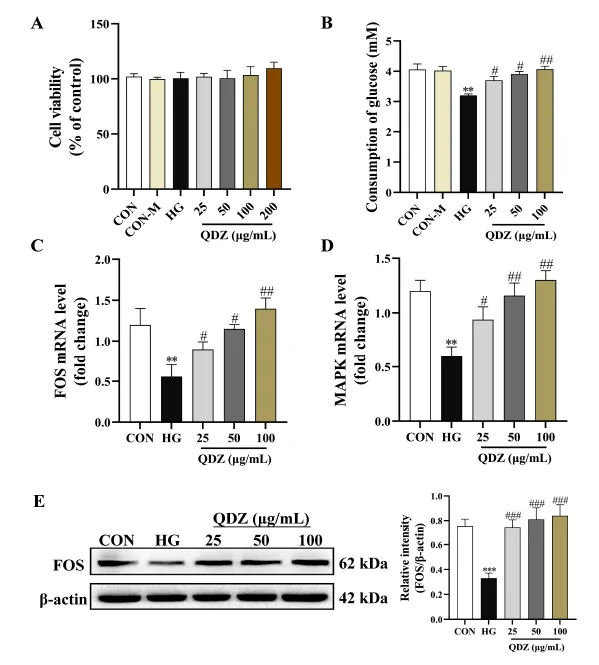
QDZ upregulates the expression of FOS and MAPK signaling pathways and promotes HG-induced glucose uptake in HEK-293 cells. **(A)** Effects of QDZ on HEK-293 cells viability; **(B)** Glucose consumption; **(C)** The expression of FOS at the mRNA level; **(D)** The expression of MAPK at the mRNA level; **(E)** The expression of FOS at the protein level. The relative intensities of proteins were normalized against β-actin. Data are shown as M±SD from three independent experiments, n=3. *P<0.05, **P<0.01, ***P<0.001 *vs*. CON group; *^#^P<0.05*, *^##^P<0.01*, *^###^P<0.001* *vs*. HG group.

**Table 1:** The degree of the active ingredient in QDZ.

**Mol ID**	**Molecule Name**	**Degree**	**Functional Activity**
MOL000098	Quercetin	107	Hepatoprotective, antidiabetic, anti-obesity, neuroprotective, cardioprotective, wound healing, antimicrobial, and immunomodulatory
MOL000422	Kaempferol	45	Anti-inflammatory, anti-cancer, antioxidant, and anti-diabetic
MOL002773	Beta-carotene	19	Antioxidant, anti-inflammatory, anti-obesity, and anti-diabetic
MOL000358	Beta-sitosterol	17	Anxiolytic, analgesic, immunomodulatory, antimicrobial, anticancer, anti - inflammatory, and antioxidant
MOL011604	Syringetin	16	Anti-osteoporosis and anti-cancer
MOL009031	Epiquinidine	15	Anti-malaria
MOL009015	(-)-Tabernemontanine	13	Anti-cancer
MOL008240	(E)-3-[4-[(1R,2R)-2-hydroxy-2-(4-hydroxy-3-methoxy-phenyl)-1-methylol-ethoxy]-3-methoxy-phenyl]acrolein	13	Unclear
MOL009055	Hirsutin	12	Anti-inflammatory, antiviral, anti-diabetic, and antioxidant
MOL007059	3-beta-Hydroxymethy llenetanshiquinone	11	Unclear
MOL009029	Dehydrodiconiferyl alcohol 4,gamma'-di-O-beta-D-glucopyanoside	11	Unclear
MOL009053	4-[(2S,3R)-5-[(E)-3-hydroxyprop-1-enyl]-7-methoxy-3-methylol-2,3-dihydrobenzofuran-2-yl]-2-methoxy-phenol	8	Unclear
MOL002058	Medioresil	7	Anti-inflammatory and antioxidant
MOL000443	Erythraline	7	Anti-cancer and anti-inflammatory
MOL009009	(+)-medioresinol	6	Antibacterial and antioxidant
MOL009047	(+)-Eudesmin	6	Anti-inflammatory, anticancer, antimicrobial, and antimalarial
MOL006709	AIDS214634	5	Unclear
MOL000073	Ent-Epicatechin	5	Antioxidant, anti-inflammatory, anticancer, and antibacterial properties
MOL004367	Olivil	4	Antioxidant, and antibacterial properties
MOL007563	Yangambin	4	Antiplatelet and anti-allergic
MOL009027	Cyclopamine	4	Anti-cancer
MOL000211	Mairin	2	Anti-fibrosis
MOL009057	Liriodendrin	2	Anti-inflammatory, antioxidant and anti-fibrosis

## Data Availability

The data and supportive information are available within the article.

## LIST OF ABBREVIATIONS

BP: Biological Processes
CC: Cellular Components
DKD: Diabetic Kidney Disease
DEGs: Differentially Expressed Genes
DL: Drug-Likeness
ECL: Enhanced Chemiluminescence
FBS: Fetal Bovine Serum
GO: Gene Ontology
GSEA: Gene Set Enrichment Analysis
GFR: Glomerular Filtration Rate
HG: High-Glucose
LG: Low-Glucose
MSigDB: Molecular Signatures Database
MF: Molecular Functions
OB: Oral Bioavailability
PVDF: Polyvinylidene Fluoride
PDB: Protein Data Bank
PPI: Protein-Protein Interaction
QDZ: Quan-du-zhong Capsule
qRT-PCR: Quantitative real-time PCR
RT: Room Temperature
STR: Short Tandem Repeat
TCM: Traditional Chinese medicine
TCMSP: Traditional Chinese Medicine Systems Pharmacology
TBST: Tris-buffered saline with Tween-20

## References

[1] Gupta S., Dominguez M., Golestaneh L. (2023). Diabetic kidney disease.. Med. Clin. North Am..

[2] Scilletta S., Di Marco M., Miano N., Filippello A., Di Mauro S., Scamporrino A., Musmeci M., Coppolino G., Di Giacomo Barbagallo F., Bosco G., Scicali R., Piro S., Purrello F., Di Pino A. (2023). Update on diabetic kidney disease (DKD): Focus on non-albuminuric DKD and cardiovascular risk.. Biomolecules.

[3] Bonner R., Albajrami O., Hudspeth J., Upadhyay A. (2020). Diabetic kidney disease.. Prim. Care.

[4] Oshima M., Shimizu M., Yamanouchi M., Toyama T., Hara A., Furuichi K., Wada T. (2021). Trajectories of kidney function in diabetes: A clinicopathological update.. Nat. Rev. Nephrol..

[5] Rayego-Mateos S., Rodrigues-Diez R.R., Fernandez-Fernandez B., Mora-Fernández C., Marchant V., Donate-Correa J., Navarro-González J.F., Ortiz A., Ruiz-Ortega M. (2023). Targeting inflammation to treat diabetic kidney disease: The road to 2030.. Kidney Int..

[6] Reidy K., Kang H.M., Hostetter T., Susztak K. (2014). Molecular mechanisms of diabetic kidney disease.. J. Clin. Invest..

[7] Han M., Li J., Wu Y., Tang Z. (2024). Potential immune-related therapeutic mechanisms of multiple traditional Chinese medicines on type 2 diabetic nephropathy based on bioinformatics, network pharmacology and molecular docking.. Int. Immunopharmacol..

[8] He X., Wang J., Li M., Hao D., Yang Y., Zhang C., He R., Tao R. (2014). Eucommia ulmoides Oliv.: Ethnopharmacology, phytochemistry and pharmacology of an important traditional Chinese medicine.. J. Ethnopharmacol..

[9] Li X., Yang W., Dai C., Qiu Z., Luan X., Zhang X., Zhang L. (2025). Integrative multi-Omics and network pharmacology reveal angiogenesis promotion by Quan-Du-Zhong Capsule *via* VEGFA/PI3K-Akt pathway.. J. Ethnopharmacol..

[10] Guo F., Zhang W., Su J., Xu H., Yang H. (2019). Prediction of drug positioning for Quan-Du-Zhong capsules against hypertensive nephropathy based on the robustness of disease network.. Front. Pharmacol..

[11] Xu X., Tian W., Duan W., Pan C., Huang M., Wang Q., Yang Q., Wen Z., Tang Y., Xiong Y., Zhu Z., Liu Y., Wei D., Qi W., Ouyang X., Ying S., Wang X., Zhou Z., Li X., Cui Y., Yang S., Xu H. (2023). Quanduzhong capsules for the treatment of grade 1 hypertension patients with low-to-moderate risk: A multicenter, randomized, double-blind, placebo-controlled clinical trial.. Front. Pharmacol..

[12] Hsieh C.C., Chang C.C., Hsu Y.C., Lin C.L. (2022). Immune modulation by myeloid-derived suppressor cells in diabetic kidney disease.. Int. J. Mol. Sci..

[13] Hsiao P.J., Kao W.Y., Sung L.C., Lin C.Y., Tsou L.L.A., Kao Y.H., Chou C.L., Lee K.T. (2025). The role of mesenchymal stem cells in treating diabetic kidney disease: Immunomodulatory effects and kidney regeneration.. Int. J. Med. Sci..

[14] Chang S.L., Patel N.A., Romero A.A., Thompson J., Zadina J.E. (1996). FOS expression induced by interleukin-1 or acute morphine treatment in the rat hypothalamus is attenuated by chronic exposure to morphine.. Brain Res..

[15] Min W., Ghosh S., Lengyel P. (1996). The interferon-inducible p202 protein as a modulator of transcription: Inhibition of NF-kappa B, c-Fos, and c-Jun activities.. Mol. Cell. Biol..

[16] Wang Q., Wang S., Cui L., Zhang Y., Waterhouse G.I.N., Sun-Waterhouse D., Ma C., Kang W. (2025). Flammulina velutipes polysaccharide exerts immunomodulatory function involving RSAD2 to regulate the NF-κB/MAPK signaling pathway in RAW264.7 macrophage cells and in mouse spleen cells.. Int. J. Biol. Macromol..

[17] Sharma M.K., Dugan E.M., Huang M.Y., Jackson C., Pataki M.J., Gracey P.R., McGovern C.J., Tako E. (2025). Dietary grape pomace ameliorates intestinal damage and oxidative stress by modulating MAPK-Nrf2/ARE pathways in coccidia-challenged broilers (Gallus gallus).. Poult. Sci..

[18] Szklarczyk D., Gable A.L., Nastou K.C., Lyon D., Kirsch R., Pyysalo S., Doncheva N.T., Legeay M., Fang T., Bork P., Jensen L.J., von Mering C. (2021). The STRING database in 2021: Customizable protein–protein networks, and functional characterization of user-uploaded gene/measurement sets.. Nucleic Acids Res..

[19] Li S., Wu X., Ma Y., Zhang H., Chen W. (2023). Prediction and verification of the active ingredients and potential targets of Erhuang Quzhi Granules on non-alcoholic fatty liver disease based on network pharmacology.. J. Ethnopharmacol..

[20] Qiu P., Mi A., Hong C., Huang S., Ma Q., Luo Q., Qiu J., Jiang H., Chen Y., Chen F., Yan H., Zhao J., Kong Y., Du Y., Li C., Kong D., Efferth T., Lou D. (2024). An integrated network pharmacology approach reveals that Ampelopsis grossedentata improves alcoholic liver disease *via* TLR4/NF-κB/MLKL pathway.. Phytomedicine.

[21] Barrett T., Wilhite S.E., Ledoux P., Evangelista C., Kim I.F., Tomashevsky M., Marshall K.A., Phillippy K.H., Sherman P.M., Holko M., Yefanov A., Lee H., Zhang N., Robertson C.L., Serova N., Davis S., Soboleva A. (2013). NCBI GEO: Archive for functional genomics data sets--Update.. Nucleic Acids Res..

[22] Pan Y., Jiang S., Hou Q., Qiu D., Shi J., Wang L., Chen Z., Zhang M., Duan A., Qin W., Zen K., Liu Z. (2018). Dissection of glomerular transcriptional profile in patients with diabetic nephropathy: SRGAP2a protects podocyte structure and function.. Diabetes.

[23] Shi J.S., Qiu D.D., Le W.B., Wang H., Li S., Lu Y.H., Jiang S. (2018). Identification of transcription regulatory relationships in diabetic nephropathy.. Chin. Med. J. (Engl.).

[24] Ritchie M.E., Phipson B., Wu D., Hu Y., Law C.W., Shi W., Smyth G.K. (2015). limma powers differential expression analyses for RNA-sequencing and microarray studies.. Nucleic Acids Res..

[25] Jovičić S.M. (2024). Analysis of total RNA as a potential biomarker of developmental neurotoxicity *in silico.*. Health Informatics J..

[26] Ou Y., Xia C., Ye C., Liu M., Jiang H., Zhu Y., Yang D. (2024). Comprehensive scRNA-seq analysis to identify new markers of M2 macrophages for predicting the prognosis of prostate cancer.. Ann. Med..

[27] Hänzelmann S., Castelo R., Guinney J. (2013). GSVA: Gene set variation analysis for microarray and RNA-Seq data.. BMC Bioinformatics.

[28] Liberzon A., Subramanian A., Pinchback R., Thorvaldsdóttir H., Tamayo P., Mesirov J.P. (2011). Molecular signatures database (MSigDB) 3.0.. Bioinformatics.

[29] Cai D., Wang J., Gao B., Li J., Wu F., Zou J.X., Xu J., Jiang Y., Zou H., Huang Z., Borowsky A.D., Bold R.J., Lara P.N., Li J.J., Chen X., Lam K.S., To K.F., Kung H.J., Fiehn O., Zhao R., Evans R.M., Chen H.W. (2019). RORγ is a targetable master regulator of cholesterol biosynthesis in a cancer subtype.. Nat. Commun..

[30] Zheng W., Lin D., Shi S., Ren J., Wu J., Wang M., Wan S. (2024). Identifying shared diagnostic genes and mechanisms in vascular dementia and Alzheimer’s disease *via* bioinformatics and machine learning.. J. Alzheimers Dis. Rep..

[31] Newman A.M., Liu C.L., Green M.R., Gentles A.J., Feng W., Xu Y., Hoang C.D., Diehn M., Alizadeh A.A. (2015). Robust enumeration of cell subsets from tissue expression profiles.. Nat. Methods.

[32] Xu X., Wu J. (2025). Elevated CXCL1 expression contributes to the development of pediatric Crohn’s disease and serves as a diagnostic marker: A reanalysis based on the integrative bioinformatics.. Cytokine.

[33] Drew H.R., Wing R.M., Takano T., Broka C., Tanaka S., Itakura K., Dickerson R.E. (1981). Structure of a B-DNA dodecamer: Conformation and dynamics.. Proc. Natl. Acad. Sci. USA.

[34] Ji L., Song T., Ge C., Wu Q., Ma L., Chen X., Chen T., Chen Q., Chen Z., Chen W. (2023). Identification of bioactive compounds and potential mechanisms of scutellariae radix-coptidis rhizoma in the treatment of atherosclerosis by integrating network pharmacology and experimental validation.. Biomed. Pharmacother..

[35] Pandarangga P., Simarmata Y.T.R.M.R., Liu A.B.H., Haryati D.A.F. (2024). *In silico* simulation of hyperoside, isoquercetin, quercetin, and quercitrin as potential antivirals against the pNP868R protein of African swine fever virus.. Vet. World.

[36] Eberhardt J., Santos-Martins D., Tillack A.F., Forli S. (2021). AutoDock Vina 1.2.0: New Docking Methods, Expanded Force Field, and Python Bindings.. J. Chem. Inf. Model..

[37] Trott O., Olson A.J. (2010). AutoDock Vina: Improving the speed and accuracy of docking with a new scoring function, efficient optimization, and multithreading.. J. Comput. Chem..

[38] Bangar N.S., Dixit A., Apte M.M., Tupe R.S. (2025). Syzygium cumini (L.) skeels mitigate diabetic nephropathy by regulating Nrf2 pathway and mitocyhondrial dysfunction: *In vitro* and *in vivo* studies.. J. Ethnopharmacol..

[39] Mohammad Ahmad (2023). Ahmad, M.; Hussein Hamzah, I. Long non-coding RNA NEAT1 inhibits high glucose-induced EMT and renal fibrogenesis in Human Embryonic Kidney 293 cells *via* regulating miR-204/SOX4 axis.. Cell. Mol. Biol..

[40] Monteiro M.B., Thieme K., Santos-Bezerra D.P., Queiroz M.S., Woronik V., Passarelli M., Machado U.F., Giannella-Neto D., Oliveira-Souza M., Corrêa-Giannella M.L. (2016). Beta-2-microglobulin (B2M) expression in the urinary sediment correlates with clinical markers of kidney disease in patients with type 1 diabetes.. Metabolism.

[41] Wang Y., Chen J., Chen C., Peng H., Lin X., Zhao Q., Chen S., Wang X. (2022). Growth differentiation factor-15 overexpression promotes cell proliferation and predicts poor prognosis in cerebral lower-grade gliomas correlated with hypoxia and glycolysis signature.. Life Sci..

[42] Zhou H., Xiao J., Cheng Q., Wang W., Peng H., Lin X., Chen J., Wang X. (2024). Metformin inhibits migration and epithelial-to-mesenchymal transition in non-small cell lung cancer cells through AMPK-mediated GDF15 induction.. Eur. J. Pharmacol..

[43] Tang Y., Li M., Wang J., Pan Y., Wu F.X. (2015). CytoNCA: A cytoscape plugin for centrality analysis and evaluation of protein interaction networks.. Biosystems.

[44] Pei J., Peng J., Wu M., Zhan X., Wang D., Zhu G., Wang W., An N., Pan X. (2025). Exploring potential targets and mechanisms of renal tissue damage caused by N-(1,3-dimethylbutyl)-N′-phenyl-p-phenylenediamine quinone (6-PPDQ) through network toxicology and animal experiments: A case of chronic kidney disease.. Sci. Total Environ..

[45] Ichisaka Y., Takei C., Naito K., Higa M., Yano S., Niwa T., Shimizu H. (2025). The role of indoxyl sulfate in exacerbating colorectal cancer during chronic kidney disease progression: Insights into the Akt/β-Catenin/c-Myc and AhR/c-Myc pathways in HCT-116 colorectal cancer cells.. Toxins (Basel).

[46] Miao J., Wei C., Wang H.L., Li Y.Q., Yu X.M., Yang X., Su H.W., Li P., Wang L. (2024). Mechanism of Chaihuang-Yishen formula to attenuate renal fibrosis in the treatment of chronic kidney disease: Insights from network pharmacology and experimental validation.. Heliyon.

[47] Fan J., Kong C., Yu B., Wang R., Qi Z. (2025). Investigation of the potential pharmacological substance basis and mechanism of action of xuantu granules in treating diabetic kidney disease based on UHPLC-Q-Exactive-HRMS and bioinformatics.. Comb. Chem. High Throughput Screen..

[48] Ansari Z., Chaurasia A. (2025). Exploring inflammatory and fibrotic mechanisms driving diabetic nephropathy progression.. Cytokine Growth Factor Rev..

[49] Li H.D., You Y.K., Shao B.Y., Wu W.F., Wang Y.F., Guo J.B., Meng X.M., Chen H. (2022). Roles and crosstalks of macrophages in diabetic nephropathy.. Front. Immunol..

[50] Yan J., Li X., Liu N., He J.C., Zhong Y. (2023). Relationship between macrophages and tissue microenvironments in diabetic kidneys.. Biomedicines.

[51] Lin J., Li X., Lin Y., Huang Z., He F., Xiong F. (2023). Unveiling *FOS* as a potential diagnostic biomarker and emetine as a prospective therapeutic agent for diabetic nephropathy.. J. Inflamm. Res..

[52] Hsin K.Y., Ghosh S., Kitano H. (2013). Combining machine learning systems and multiple docking simulation packages to improve docking prediction reliability for network pharmacology.. PLoS One.

[53] Younossi Z.M., Koenig A.B., Abdelatif D., Fazel Y., Henry L., Wymer M. (2016). Global epidemiology of nonalcoholic fatty liver disease—Meta‐analytic assessment of prevalence, incidence, and outcomes.. Hepatology.

[54] Bae J., Won Y.J., Lee B.W. (2021). Non-Albumin Proteinuria (NAP) as a complementary marker for diabetic kidney disease (DKD).. Life.

[55] Peng Q.Y., An Y., Jiang Z.Z., Xu Y. (2024). The role of immune cells in DKD: Mechanisms and targeted therapies.. J. Inflamm. Res..

[56] Abdou H.M., Abd Elkader H.T.A.E. (2022). The potential therapeutic effects of Trifolium alexandrinum extract, hesperetin and quercetin against diabetic nephropathy *via* attenuation of oxidative stress, inflammation, GSK-3β and apoptosis in male rats.. Chem. Biol. Interact..

[57] Anjaneyulu M., Chopra K. (2004). Quercetin, an anti-oxidant bioflavonoid, attenuates diabetic nephropathy in rats.. Clin. Exp. Pharmacol. Physiol..

[58] Alshehri A.S. (2023). Kaempferol attenuates diabetic nephropathy in streptozotocin-induced diabetic rats by a hypoglycaemic effect and concomitant activation of the Nrf-2/Ho-1/antioxidants axis.. Arch. Physiol. Biochem..

[59] Yang S., Zhang Y., Zheng C. (2024). β-Sitosterol mitigates apoptosis, oxidative stress and inflammatory response by inactivating TLR4/NF-кB pathway in cell models of diabetic nephropathy.. Cell Biochem. Biophys..

[60] Hickson L.J., Langhi Prata L.G.P., Bobart S.A., Evans T.K., Giorgadze N., Hashmi S.K., Herrmann S.M., Jensen M.D., Jia Q., Jordan K.L., Kellogg T.A., Khosla S., Koerber D.M., Lagnado A.B., Lawson D.K., LeBrasseur N.K., Lerman L.O., McDonald K.M., McKenzie T.J., Passos J.F., Pignolo R.J., Pirtskhalava T., Saadiq I.M., Schaefer K.K., Textor S.C., Victorelli S.G., Volkman T.L., Xue A., Wentworth M.A., Wissler Gerdes E.O., Allison D.B., Dickinson S.L., Ejima K., Atkinson E.J., Lenburg M., Zhu Y., Tchkonia T., Kirkland J.L. (2020). Corrigendum to ‘Senolytics decrease senescent cells in humans: Preliminary report from a clinical trial of Dasatinib plus Quercetin in individuals with diabetic kidney disease’ EBioMedicine 47 (2019) 446–456.. EBioMedicine.

[61] Liu F., Feng Q., Yang M., Yang Y., Nie J., Wang S. (2024). Quercetin prevented diabetic nephropathy by inhibiting renal tubular epithelial cell apoptosis *via* the PI3K / AKT pathway.. Phytother. Res..

[62] El-Marasy S.A., Farouk H., Khattab M.S., Moustafa P.E. (2024). Beta-carotene ameliorates diabetic nephropathy in rats: Involvement of AMPK/SIRT1/autophagy pathway.. Immunopharmacol. Immunotoxicol..

[63] Chmiel M., Stompor-Gorący M. (2022). The spectrum of pharmacological actions of syringetin and its natural derivatives—A summary review.. Nutrients.

[64] Wu B., Song H.P., Zhou X., Liu X.G., Gao W., Dong X., Li H.J., Li P., Yang H. (2016). Screening of minor bioactive compounds from herbal medicines by *in silico* docking and the trace peak exposure methods.. J. Chromatogr. A.

[65] Yap E.L., Pettit N.L., Davis C.P., Nagy M.A., Harmin D.A., Golden E., Dagliyan O., Lin C., Rudolph S., Sharma N., Griffith E.C., Harvey C.D., Greenberg M.E. (2021). Bidirectional perisomatic inhibitory plasticity of a Fos neuronal network.. Nature.

[66] Bai L., Mao R., Wang J., Ding L., Jiang S., Gao C., Kang H., Chen X., Sun X., Xu J. (2015). ERK1/2 promoted proliferation and inhibited apoptosis of human cervical cancer cells and regulated the expression of c-Fos and c-Jun proteins.. Med. Oncol..

[67] Chan Q.K.Y., Lam H-M., Ng C-F., Lee A.Y.Y., Chan E.S.Y., Ng H-K., Ho S-M., Lau K-M. (2010). Activation of GPR30 inhibits the growth of prostate cancer cells through sustained activation of Erk1/2, c-jun/c-fos-dependent upregulation of p21, and induction of G2 cell-cycle arrest.. Cell Death Differ..

[68] Malik S., Suchal K., Khan S.I., Bhatia J., Kishore K., Dinda A.K., Arya D.S. (2017). Apigenin ameliorates streptozotocin-induced diabetic nephropathy in rats *via* MAPK-NF-κB-TNF-α and TGF-β1-MAPK-fibronectin pathways.. Am. J. Physiol. Renal Physiol..

[69] Mao Z.M., Shen S.M., Wan Y.G., Sun W., Chen H.L., Huang M.M., Yang J.J., Wu W., Tang H.T., Tang R.M. (2015). Huangkui capsule attenuates renal fibrosis in diabetic nephropathy rats through regulating oxidative stress and p38MAPK/Akt pathways, compared to α-lipoic acid.. J. Ethnopharmacol..

[70] Chen Y., Peng Y., Li P., Jiang Y., Song D. (2024). Ginsenoside Rg3 induces mesangial cells proliferation and attenuates apoptosis by miR-216a-5p/MAPK pathway in diabetic kidney disease.. Aging (Albany NY).

[71] Choi R.J., Mohamad Zobir S.Z., Alexander-Dann B., Sharma N., Ma M.K.L., Lam B.Y.H., Yeo G.S.H., Zhang W., Fan T.P., Bender A. (2021). Combination of ginsenosides Rb2 and Rg3 promotes angiogenic phenotype of human endothelial cells *via* PI3K/Akt and MAPK/ERK pathways.. Front. Pharmacol..

[72] Lee Y., Park A., Park Y.J., Jung H., Kim T.D., Noh J.Y., Choi I., Lee S., Ran Yoon S. (2022). Ginsenoside 20(R)-Rg3 enhances natural killer cell activity by increasing activating receptor expression through the MAPK/ERK signaling pathway.. Int. Immunopharmacol..

[73] Talukdar S., Oh D.Y., Bandyopadhyay G., Li D., Xu J., McNelis J., Lu M., Li P., Yan Q., Zhu Y., Ofrecio J., Lin M., Brenner M.B., Olefsky J.M. (2012). Neutrophils mediate insulin resistance in mice fed a high-fat diet through secreted elastase.. Nat. Med..

[74] Herrero-Cervera A., Soehnlein O., Kenne E. (2022). Neutrophils in chronic inflammatory diseases.. Cell. Mol. Immunol..

[75] Wierusz-Wysocka B., Wykretowicz A., Byks H., Sadurska K., Wysocki H. (1993). Polymorphonuclear neutrophils adherence, superoxide anion (O2−) production and HBA1 level in diabetic patients.. Diabetes Res. Clin. Pract..

